# Supplementary material for: Sex-related differences in pre-dialysis trajectories and dialysis initiation: A French nationwide retrospective study
Source: PLoS One. 2024 Mar 27;19(3):e0299601. doi: 10.1371/journal.pone.0299601 (PMC10971748; doi:10.1371/journal.pone.0299601)
Supplement: S1 File — (PDF) [file pone.0299601.s001.pdf]

Supplemental material

<u>Table of content Supplemental material</u>

S1 Table: Classification of the primary kidney diseases in the REIN registry according to their progression

S2 Table: Diagnostic codes from the International Classification of Diseases, Tenth Revision (ICD-10) used to identify nephrology-related hospital stays (with the exception of preparatory care for dialysis)

S3 Table: Frequencies of consultations with other medical specialties in the 2 years before dialysis start (ordered by decreasingfrequencies of consultations by men)

S4 Table. Characteristics at dialysis initiation and healthcare utilization in the year before dialysis start of incident patients in France in2015, stratified in function of the dialysis start context (Planned vs EDS) and sex

S1 Table: Classification of the primary kidney diseases in the REIN registry according to their progression

**Acute nephropathy (including CKD exacerbation/flare)**	**Slowly progressive nephropathy**	**Uncertain/Variable progression**
Amyloidosis	Kidney agenesis, hypoplasia, dysplasia	Glomerulonephritis without histological examination
Extracapillary glomerulonephritis	Morphological abnormality	Glomerulonephritis with histological diagnosis
Cryoglobulinemia	Diabetes	Secondary glomerular nephritis
Wegener's granulomatosis	Glomerulonephritis with focal segmental glomerulosclerosis	Unknown
Acute kidney injury	Membranous glomerulonephritis	Vascular nephropathy from other cause
Other systemic disease	Membranoproliferative glomerulonephritis	Vascular nephropathy, unprecise cause
Myeloma	Kidney infection	
Ischemic nephropathy, cholesterol-embolization syndrome	Hereditary kidney disease	
Lupus nephropathy	Tubulointerstitial nephritis due to neurological bladder	
Vascular nephropathy due to malignant hypertension	Tubulointerstitial nephritis secondary to lithiasis	
Traumatic loss of kidney	Tubulointerstitial nephritis due to acquired obstructive uropathy	
Schönlein-Henoch purpura	Tubulointerstitial nephritis due to congenital obstructive uropathy	
Systemic scleroderma	Other tubulointerstitial nephritis	
Goodpasture's syndrome	Nephrocalcinosis	
Thrombotic microangiopathy	CKD due to vesicoureteral reflux	
Toxic nephropathy	Hereditary nephropathy with deafness	
	Vascular nephropathy due to hypertension	
	IgA nephropathy	
	Primary oxalosis	
	Cystic disease	
	Polycystic kidney	
	Renal tuberculosis	
	Tubulopathies (Dent, Lowe, Barter…)	
	Renal or urinary tract cancer	

S2 Table: Diagnostic codes from the International Classification of Diseases, Tenth Revision (ICD-10) used to identify nephrology-related hospital stays (with the exception of preparatory care for dialysis)

**Diagnostic code**	Description
N00-N08	Glomerular diseases
N10-N16	Renal tubulo-interstitial diseases
N17-N19	Renal failure
N20-N23	Urolithiasis
N25-N29	Other disorders of kidney and ureter

S3 Table. Frequencies of consultations with other medical specialties in the 2 years before dialysis start (ordered by decreasing frequencies of consultations by men)

	Men				Women			
Medical specialty	Number of consultations	**Ratio / 100 patients**	Number of patients with ≥ 1 consultation	**% (N=5695)**	Number of consultations	**Ratio / 100 patients**	Number of patients with ≥ 1 consultation	**% (N=3161)**
Anesthesiology	3933	**69.1**	2642	**46.4**	2208	**69.9**	1435	**45.4**
Ophthalmology	3731	**65.5**	1934	**34.0**	2202	**69.7**	1121	**35.5**
Urologic surgery	3379	**59.3**	1445	**25.4**	858	**27.1**	399	**12.6**
Endocrinology	3081	**54.1**	900	**15.8**	1621	**51.3**	544	**17.2**
Vascular surgery	2965	**52.1**	1369	**24.0**	1480	**46.8**	659	**20.8**
Cardiology	2855	**50.1**	1492	**26.2**	1382	**43.7**	772	**24.4**
General surgery	2028	**35.6**	960	**16.9**	1223	**38.7**	531	**16.8**
Internal medicine	1846	**32.4**	541	**9.5**	1077	**34.1**	313	**9.9**
Dermatology	1664	**29.2**	907	**15.9**	774	**24.5**	421	**13.3**
Dental surgery	1468	**25.8**	1073	**18.8**	810	**25.6**	578	**18.3**
Gastroenterology and hepatology	1381	**24.2**	744	**13.1**	688	**21.8**	401	**12.7**
Pulmonology	1172	**20.6**	582	**10.2**	526	**16.6**	248	**7.8**
Orthopedic surgery and traumatology	1170	**20.5**	522	**9.2**	878	**27.8**	366	**11.6**
Otolaryngology	1152	**20.2**	661	**11.6**	498	**15.8**	328	**10.4**
Psychiatry	1047	**18.4**	164	**2.9**	1341	**42.4**	126	**4.0**
Cardio-vascular surgery	893	**15.7**	463	**8.1**	482	**15.2**	239	**7.6**
Rheumatology	761	**13.4**	380	**6.7**	603	**19.1**	289	**9.1**
Hematology	680	**11.9**	200	**3.5**	359	**11.4**	111	**3.5**
Visceral and digestive surgery	596	**10.5**	328	**5.8**	296	**9.4**	162	**5.1**
Oncology	501	**8.8**	160	**2.8**	440	**13.9**	100	**3.2**
Neurology	370	**6.5**	216	**3.8**	128	**4.0**	73	**2.3**
Physical medicine and rehabilitation	313	**5.5**	108	**1.9**	93	**2.9**	42	**1.3**
Radiation oncology	212	**3.7**	79	**1.4**	72	**2.3**	32	**1.0**
Oral and maxillofacial surgery	124	**2.2**	95	**1.7**	70	**2.2**	51	**1.6**
Radiotherapy	115	**2.0**	58	**1.0**	119	**3.8**	39	**1.2**
Plastic surgery	93	**1.6**	53	**0.9**	69	**2.2**	37	**1.2**
Geriatrics	79	**1.4**	26	**0.5**	37	**1.2**	18	**0.6**
Neuropsychiatry	77	**1.4**	45	**0.8**	38	**1.2**	19	**0.6**
Neurological surgery	71	**1.2**	44	**0.8**	56	**1.8**	38	**1.2**
Pediatrics	45	**0.8**	19	**0.3**	64	**2.0**	14	**0.4**
Obstetrics and gynecology	34	**0.6**	26	**0.4**	807	**25.5**	514	**16.3**
Midwife	1	**0**	1	**0**	40	**1.3**	15	**0.5**

S4 Table. Characteristics at dialysis initiation and healthcare utilization in the year before dialysis start of incident patients in France in2015, stratified in function of the dialysis start context (Planned vs EDS) and sex

	Planned Dialysis Start		Emergency Dialysis Start	
	Men n=3933	Women n=2242	Men n=1762	Women n=919
**Age, median [Q1-Q3]**	71.4 [61.0, 80.1]	71.1 [59.7, 80.2]	70.5 [60.6, 79.7]	72.1 [60.3, 80.8]
18-45 years	299 (7.6%)	187 (8.3%)	168 (9.5%)	82 (8.9%)
45-60 years	608 (15.5%)	380 (16.9%)	257 (14.6%)	143 (15.6%)
60-75 years	1439 (36.6%)	760 (33.9%)	655 (37.2%)	304 (33.1%)
≥ 75 years	1587 (40.4%)	915 (40.8%)	682 (38.7%)	390 (42.4%)
**Initial kidney disease (nephropathy)**				
Polycystic	200 (5.1%)	194 (8.7%)	32 (1.8%)	26 (2.8%)
Diabetic	896 (22.8%)	528 (23.6%)	414 (23.5%)	226 (24.6%)
Vascular and hypertensive	1160 (29.5%)	540 (24.1%)	470 (26.7%)	208 (22.6%)
Glomerulonephritis	523 (13.3%)	228 (10.2%)	224 (12.7%)	106 (11.5%)
Pyelonephritis	151 (3.8%)	102 (4.5%)	81 (4.6%)	33 (3.6%)
Others and unknown	1003 (25.5%)	650 (29.0%)	541 (30.7%)	320 (34.8%)
**Nephropathy progression type**				
Slowly progressive	2792 (71.0%)	1535 (68.5%)	1108 (62.9%)	528 (57.5%)
Acute	387 (9.8%)	266 (11.9%)	254 (14.4%)	169 (18.4%)
Variable/uncertain	754 (19.2%)	441 (19.7%)	400 (22.7%)	222 (24.2%)
**Body mass index (kg/m²)**				
< 18.5	70 (1.8%)	78 (3.5%)	50 (2.8%)	45 (4.9%)
18.5-23	738 (18.8%)	440 (19.6%)	432 (24.5%)	182 (19.8%)
23-25	587 (14.9%)	248 (11.1%)	224 (12.7%)	93 (10.1%)
25-30	1218 (31.0%)	517 (23.1%)	433 (24.6%)	201 (21.9%)
≥ 30	734 (18.7%)	606 (27.0%)	278 (15.8%)	201 (21.9%)
Missing	586 (14.9%)	353 (15.7%)	345 (19.6%)	197 (21.4%)
**Serum albumin (g/L)**				
≥ 30	2924 (74.3%)	1614 (72.0%)	1061 (60.2%)	530 (57.7%)
< 30	542 (13.8%)	324 (14.5%)	445 (25.3%)	246 (26.8%)
Missing	467 (11.9%)	304 (13.6%)	256 (14.5%)	143 (15.6%)
**Hemoglobin (g/L)**				
< 10	1851 (47.1%)	1167 (52.1%)	1123 (63.7%)	619 (67.4%)
10-12	1304 (33.2%)	708 (31.6%)	394 (22.4%)	191 (20.8%)
≥ 12	652 (16.6%)	287 (12.8%)	166 (9.4%)	63 (6.9%)
Missing	126 (3.2%)	80 (3.6%)	79 (4.5%)	46 (5.0%)
**Number of cardiovascular diseases***				
0	1655 (42.1%)	1267 (56.5%)	610 (34.6%)	411 (44.7%)

1	994 (25.3%)	521 (23.2%)	407 (23.1%)	236 (25.7%)
2	663 (16.9%)	292 (13.0%)	352 (20.0%)	150 (16.3%)
≥ 3	621 (15.8%)	162 (7.2%)	393 (22.3%)	122 (13.3%)
**Diabetes**	1753 (44.6%)	974 (43.4%)	832 (47.2%)	427 (46.5%)
Missing	18 (0.5%)	11 (0.5%)	8 (0.5%)	3 (0.3%)
**Chronic respiratory disease**	554 (14.1%)	200 (8.9%)	332 (18.8%)	106 (11.5%)
Missing	107 (2.7%)	62 (2.8%)	66 (3.7%)	25 (2.7%)
**Mobility**				
Walk without help	3202 (81.4%)	1754 (78.2%)	1236 (70.1%)	604 (65.7%)
Need assistance	317 (8.1%)	254 (11.3%)	244 (13.8%)	148 (16.1%)
Totally dependent	114 (2.9%)	79 (3.5%)	111 (6.3%)	75 (8.2%)
Missing	300 (7.6%)	155 (6.9%)	171 (9.7%)	92 (10.0%)
**Dialysis modality**				
Hemodialysis	3462 (88.0%)	1957 (87.3%)	1726 (98.0%)	899 (97.8%)
Peritoneal dialysis	471 (12.0%)	285 (12.7%)	36 (2.0%)	20 (2.2%)
**eGFR (ml/min per 1.73m², CKD-EPI)**				
**Median [Q1, Q3]**	8.51 [6.51, 11.1]	7.90 [6.03, 10.2]	7.76 [5.23, 11.0]	6.99 [4.91, 9.94]
Missing	353 (9.0%)	213 (9.5%)	193 (11.0%)	108 (11.8%)
**Consultations with a GP****				
0	247 (6.3%)	129 (5.8%)	174 (9.9%)	69 (7.5%)
1-7	1787 (45.4%)	916 (40.9%)	765 (43.4%)	370 (40.3%)
> 8	1899 (48.3%)	1197 (53.4%)	823 (46.7%)	480 (52.2%)
**Consultations with a nephrologist****				
0	666 (16.9%)	388 (17.3%)	721 (40.9%)	408 (44.4%)
1-2	697 (17.7%)	394 (17.6%)	412 (23.4%)	203 (22.1%)
≥ 3	2570 (65.3%)	1460 (65.1%)	629 (35.7%)	308 (33.5%)
**Nephrology-related hospitalizations****				
**0**	1676 (42.6%)	953 (42.5%)	786 (44.6%)	402 (43.7%)
1	858 (21.8%)	466 (20.8%)	315 (17.9%)	160 (17.4%)
≥ 2	1399 (35.6%)	823 (36.7%)	661 (37.5%)	357 (38.8%)
**Nephrology-unrelated hospitalizations (all other causes)****				
**0**	3094 (78.7%)	1794 (80.0%)	1301 (73.8%)	653 (71.1%)
1	495 (12.6%)	281 (12.5%)	239 (13.6%)	143 (15.6%)
≥ 2	344 (8.7%)	167 (7.4%)	222 (12.6%)	123 (13.4%)
**Preparation to dialysis-related hospitalizations****				
**0**	1930 (49.1%)	1118 (49.9%)	1393 (79.1%)	709 (77.1%)
1	1548 (39.4%)	781 (34.8%)	291 (16.5%)	145 (15.8%)
≥ 2	455 (11.6%)	343 (15.3%)	78 (4.4%)	65 (7.1%)

**<24-hour hospitalizations****				
**0**	2474 (62.9%)	1408 (62.8%)	1213 (68.8%)	618 (67.2%)
1	827 (21.0%)	404 (18.0%)	332 (18.8%)	157 (17.1%)
≥ 2	632 (16.1%)	430 (19.2%)	217 (12.3%)	144 (15.7%)
